# Uncovering waterlogging-responsive genes in cucumber through machine learning and differential gene correlation analysis

**DOI:** 10.1186/s40529-024-00433-z

**Published:** 2024-08-14

**Authors:** Zahra Zinati, Leyla Nazari, Ali Niazi

**Affiliations:** 1 Department of Agroecology, College of Agriculture and Natural Resources of Darab, Shiraz University, Shiraz, Iran; 2 Crop and Horticultural Science Research Department, Fars Agricultural and Natural Resources Research and Education Center, Agricultural Research, Education and Extension Organization (AREEO), Shiraz, Iran; 3 Institute of Biotechnology, School of Agriculture, Shiraz University, Shiraz, Iran

**Keywords:** Abiotic stress, Attribute weighting algorithms, Cucumber, Gene expression

## Abstract

As climate change intensifies, the frequency and severity of waterlogging are expected to increase, necessitating a deeper understanding of the cucumber response to this stress. In this study, three public RNA-seq datasets (PRJNA799460, PRJNA844418, and PRJNA678740) comprising 36 samples were analyzed. Various feature selection algorithms including Uncertainty, Relief, SVM (Support Vector Machine), Correlation, and logistic least absolute shrinkage, and selection operator (LASSO) were performed to identify the most significant genes related to the waterlogging stress response. These feature selection techniques, which have different characteristics, were used to reduce the complexity of the data and thereby identify the most significant genes related to the waterlogging stress response. Uncertainty, Relief, SVM, Correlation, and LASSO identified 4, 4, 10, 21, and 13 genes, respectively. Differential gene correlation analysis (DGCA) focusing on the 36 selected genes identified changes in correlation patterns between the selected genes under waterlogged versus control conditions, providing deeper insights into the regulatory networks and interactions among the selected genes. DGCA revealed significant changes in the correlation of 13 genes between control and waterlogging conditions. Finally, we validated 13 genes using the Random Forest (RF) classifier, which achieved 100% accuracy and a 1.0 Area Under the Curve (AUC) score. The SHapley Additive exPlanations (SHAP) values clearly showed the significant impact of LOC101209599, LOC101217277, and LOC101216320 on the model’s predictive power. In addition, we employed the Boruta as a wrapper feature selection method to further validate our gene selection strategy. Eight of the 13 genes were common across the four feature weighting algorithms, LASSO, DGCA, and Boruta, underscoring the robustness and reliability of our gene selection strategy. Notably, the genes LOC101209599, LOC101217277, and LOC101216320 were among genes identified by multiple feature selection methods from different categories (filtering, wrapper, and embedded). Pathways associated with these specific genes play a pivotal role in regulating stress tolerance, root development, nutrient absorption, sugar metabolism, gene expression, protein degradation, and calcium signaling. These intricate regulatory mechanisms are crucial for cucumbers to adapt effectively to waterlogging conditions. These findings provide valuable insights for uncovering targets in breeding new cucumber varieties with enhanced stress tolerance.

**Supplementary Information:**

The online version contains supplementary material available at 10.1186/s40529-024-00433-z.

## Introduction

Waterlogging is a critical environmental stress that impacts approximately 12% of the world’s arable land, resulting in significant crop yield losses, estimated at around 20% (Setter and Waters 2003). Due to ongoing global climate change, soil waterlogging is expected to increase, particularly in irrigated regions and during episodes of intense and irregular rainfall (Tian et al. 2021). Waterlogging stress presents a unique challenge for cucumber plants, despite their ability to form adventitious roots that aid gas diffusion and enhance survival in low oxygen conditions (Barickman et al. 2019; Qi et al. 2019). However, the precise physiological mechanisms governing the response of cucumber plants to waterlogging stress are still poorly understood (Olorunwa et al. 2022a). The induced morphological changes and shifts in photosynthesis and key metabolites have further highlighted the sensitivity of cucumber plants to waterlogging (Barickman et al. 2019; Olorunwa et al. 2022a, b). Consequently, developing tolerance to waterlogging stress in cucumber is an important area of research, with investigations into the underlying physiological mechanisms being paramount (Olorunwa et al. 2022a). In this context, meta-analysis has emerged as a valuable tool in plant breeding as it identifies common patterns and trends across multiple studies, providing effective information on breeding strategies (Zhang 2017). Although no specific meta-analysis has been conducted on waterlogging stress in cucumber plants, our current study provides insights into the genes and pathways involved in cucumber’s tolerance to waterlogging stress. Direct data merging involves combining the raw data from individual studies into a single dataset. It is often used in joint analyses of high-dimensional gene expression data (Krepel et al. 2022). The major concern with the direct data merging approach is heterogeneity across studies (batch effects). Integrating transcriptomic data from multiple studies using supervised machine learning models can be a powerful approach to capturing common biological signals while preserving the generalizability of the model, leading to more robust and reliable predictions (Maj et al. 2019; Pashaiasl et al. 2016).

Feature selection is a crucial step in biological data analysis, aimed at reducing dimensionality and identifying significant genes. This process can be broadly categorized into three methods: filtering, wrapper, and embedded. Filtering methods evaluate features based on their intrinsic properties, such as correlation or statistical metrics, without involving learning algorithms, making them computationally efficient but potentially overlooking feature interactions. Wrapper methods, on the other hand, utilize predictive models to assess different combinations of features, capturing interactions between features and often yielding high accuracy, although they are computationally intensive (Saeys et al. 2007). An example of a wrapper method is Boruta. Previous studies (Pashaei et al., 2019; Pashaei 2022) have shown that wrapper-based methods are highly effective for feature selection in genetic and genomic analyses. Embedded methods perform feature selection during model training, balancing efficiency with the ability to handle feature interactions while at the same time being far less computationally intensive than wrapper methods, as seen in techniques such as LASSO (Least Absolute Shrinkage and Selection Operator) and tree-based methods such as Random Forest (RF) (Saeys et al. 2007).

Supervised machine learning models, in particular various attribute weighting algorithms, have been used in gene selection. These algorithms encompass weight by principle component analysis, information gain, correlation, rule, information gain ratio, chi-squared statistic, gini index, deviation, relief, and uncertainty (Karami et al. 2019).

To delve deeper into the regulatory networks and key genes involved in the waterlogging response of cucumber, we used the powerful technique of LASSO regression to analyze high-dimensional transcriptomic data (Xiong et al. 2019).

LASSO regression has been shown to be instrumental in identifying critical genes and regulatory networks in plant gene expression data, significantly improving our understanding of plant biology and plant breeding programs (Liu et al. 2011). In rice studies, LASSO regression has successfully uncovered key gene interactions associated with salt tolerance phenotypes (Du et al. 2018), while in Arabidopsis thaliana, it was used to detect novel candidates associated with mucilage and pectin metabolism genes (Vasilevski et al. 2012). Furthermore, LASSO regression has been used to infer gene regulatory networks based on gene expression data in different ecotypes of Arabidopsis exposed to spaceflight microgravity conditions (Manian et al. 2021). In addition, transcriptome analysis has been extensively used to study the gene expression changes in cucumber plants, shedding light on key regulatory events and molecular responses (Ando et al. 2012). By analyzing the regulatory relationships between genes, differential gene correlation analysis can help identify genes responsible for specific traits, which can be used in breeding programs to develop new plant varieties with desired traits (Cui et al. 2021). By integrating various machine learning algorithms with meta-analysis, the complexity and heterogeneity of data can be effectively handled, thereby significantly improving the robustness and accuracy of the analysis (Panahi et al. 2021). In this investigation, we conducted a comprehensive analysis of three transcriptomic datasets to investigate the effects of waterlogging stress on cucumber plants. By integrating meta-analysis and machine learning techniques, we identified potential candidate genes in the response of cucumber plants to waterlogging stress. In addition, the differential gene correlation study allowed us to focus on key genes with significant roles in the adaptation of cucumbers to waterlogging stress. By gaining a deeper understanding of the molecular mechanisms and key genes governing the response to waterlogging stress in cucumber, this study provides valuable insights for future breeding programs aimed at developing waterlogging tolerant cucumber varieties, thereby contributing to ensuring global food security in the face of waterlogging under changing climatic conditions.

Our research presents an innovative integration of multiple feature selection methods from different categories (filtering, wrapper, and embedded) to comprehensively analyze the response of cucumbers to waterlogging stress. By combining these methods, we leverage their respective strengths to achieve a more robust and comprehensive gene selection process, reducing bias. Additionally, we performed a meta-analysis of three independent transcriptome datasets (PRJNA799460, PRJNA844418, and PRJNA678740), which improves the generalizability and robustness of our results by accounting for variability under different experimental conditions. Applying Differential Gene Correlation Analysis (DGCA) to the genes selected using this multi-method approach revealed new insights into the regulatory networks and interactions critical for the adaptation of cucumbers to waterlogging stress. It enabled a deeper understanding beyond merely listing differentially expressed genes. The significance of the identified genes was validated using the RF model, which achieved an accuracy of 100% and an AUC score of 1.0. SHAP values were used to interpret the model, highlighting the functional importance of genes in the waterlogging response. These genes provide valuable targets for future breeding programs to improve stress tolerance in cucumbers. We also employed the Boruta algorithm as a wrapper-based feature selection method to further validate our gene selection strategy. The substantial overlap in the identified genes across these different approaches underscores the robustness and reliability of our gene selection strategy.

## Materials and methods

**Fig. 1:**
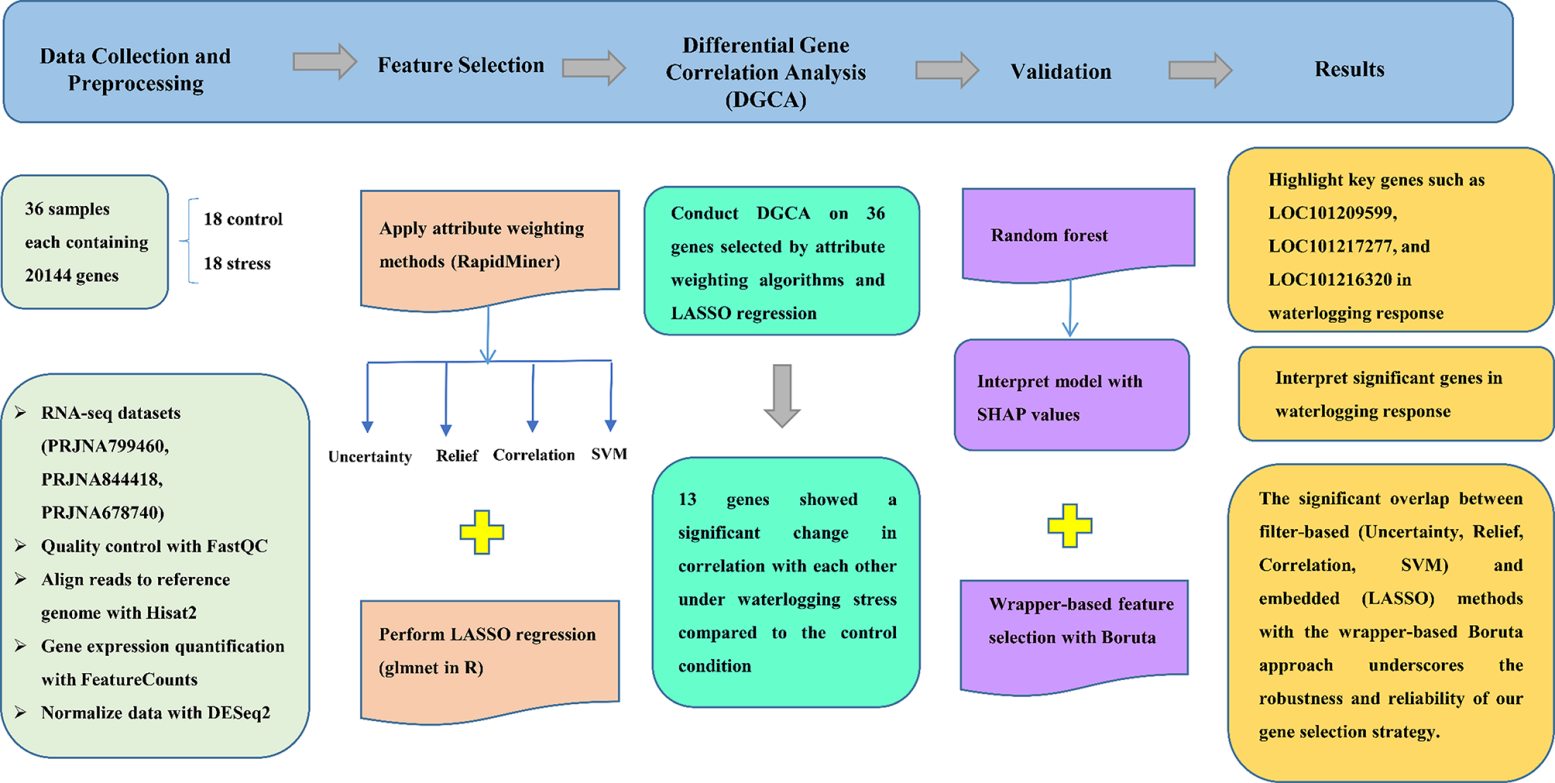
Flowchart of the study to find the key genes involved in the waterlogging stress response in cucumber

The flowchart of the study to identify the key genes involved in the waterlogging stress response in cucumber is presented in Fig. 1.

### Data information

In this research, we used three datasets from SRA (https://www.ncbi.nlm.nih.gov/sra), including PRJNA799460 (6 samples), PRJNA844418 (18 samples), and PRJNA678740 (12 samples) (Table 1).

**Table 1:** The transcriptomic raw data derived from studies investigating waterlogging stress in cucumber served as the primary dataset for the present analysis

Accession	Reference	Website	Project samples	Treatment samples	Treatment sample information	Control samples	Control sample information
PRJNA678740	Kęska et al. 2021	https://www.ncbi.nlm.nih.gov/bioproject/?term=PRJNA678740	12	3	DH2 1xH	3	DH2 Ctrl
				3	DH4 1xH	3	DH4 Ctr
PRJNA799460		https://www.ncbi.nlm.nih.gov/bioproject/?term=PRJNA799460	6	3	WL	3	CK
PRJNA844418		https://www.ncbi.nlm.nih.gov/bioproject/?term=PRJNA844418	18	3	Hypocotyl basic tissue of Zaoer-N(ME) cucumber under waterlogging stress for 48 h	3	Hypocotyl basal tissue of Zaoer-N(CME) cucumber control treatment for 48 h
				3	Hypocotyl vascular bundle of Zaoer-N(VB) cucumber under waterlogging stress for 48 h	3	Hypocotyl vascular bundles treated with Zaoer-N(CVB) cucumber control for 48 h
				3	Hypocotyl epidermis of Zaoer-N(SK) cucumber under waterlogging stress for 48 h	3	Hypocotyl epidermis of Zaoer-N (CSK) cucumber control treated for 48 h

### Quality control and mapping

We used FastQC version 0.11.4 (Andrews 2010) to assess the quality of the RNA-seq data. FastQC results indicated that there was no need to pre-process or trim the original expression data, as the quality of the raw sequencing data submitted to SRA was suitable for further analysis.

The reference genome for *Cucumis sativus* was obtained from the EnsemblPlants database in both FASTA and GFF formats. Quality-controlled reads were then aligned to the reference genome sequence of *Cucumis sativus* using Hisat2 (Kim et al. 2015).

### Gene expression quantification

In this research, reading counts were performed using FeatureCounts. In addition, Hisat2 BAM files were used as input aligned files. The RNA-seq data used in this study were unstranded, as indicated by information from studies in the SRA database. Therefore, we chose to count unstranded reads and set the feature type to ‘exon’ to ensure that only lines in the provided GTF annotation file that matched the corresponding exon were counted.

### Normalization

Normalization is a critical step in accurately comparing samples. The count data obtained from FeatureCounts was normalized using DESeq2 (Love et al. 2014). DESeq2 median ratios are an appropriate choice for differential expression analyses and gene count comparisons between samples (Evans et al. 2018). This normalization approach takes into account the components of RNA composition and sequencing depth. According to the median ratio of gene counts relevant to the geometric mean per gene, the counts are divided by the sample size specified for each sample (Anders and Huber 2010).

### Attribute weighting approaches

Attribute weighting methods were used to identify genes that discriminate between waterlogged and control conditions. The subject feature, classified as “stress” and “control,” was used as the target or label variable. In addition, the normalized expression values of the genes were used as attributes and categorized as continuous data. The resulting dataset was then imported into RapidMiner Studio software (RapidMiner 7.0.001 Gmbh). The dataset is available in a supplementary file, sheet S1.

To decrease the complexity of the data and pinpoint the most critical genes linked to the response to waterlogging stress, we employed four distinct attribute weighting methods: Uncertainty, Relief, Correlation, and SVM, using a threshold of 0.90. A value nearing 1 indicates that a particular gene plays a more significant role in discriminating between controls and waterlogging stress conditions. The genes were considered to be the primary differentiating genes. The contextual information about the attribute weighting techniques is provided as follows (RapidMiner Studio 7.6, https://docs.rapidminer.com):

### Uncertainty

The Weight by Uncertainty operator determines the weight of the attribute based on the label attribute, using the symmetric uncertainty concerning the class. Increasing the weight of the attribute increases its relevance. The following formula is used to calculate relevance:

$$\eqalign{ Relevance{\rm{ }} = & {\rm{ }}2{\rm{ }}*{\rm{ }}\left( {P\left( {Class} \right){\rm{ }} - {\rm{ }}P\left( {Class{\rm{ }}|{\rm{ }}Attribute} \right)} \right) \cr {\rm{ }} & /{\rm{ }}P\left( {Class} \right){\rm{ }} + {\rm{ }}P\left( {Attribute} \right) \cr}$$

### Relief

Relief stands out as the most important feature quality evaluation algorithm due to its simplicity and efficiency. The basic concept of Relief is to measure feature quality by assessing how well instances of the same and different classes can be distinguished when they are adjacent to each other. By using sampling examples and comparing the current feature value with the nearest examples from both classes, Relief determines the relevance of features. The resulting weights can be normalized to a range of 0 to 1 by enabling the Normalized Weights parameters.

### Correlation

By computing the correlation coefficient between two numerical variables, the correlation algorithm determines their statistical link. Pearson’s correlation coefficient, which measures the linear relationship between two variables, is the most widely used correlation coefficient. It has a range from − 1 to 1, with a value of -1 representing a perfect negative correlation, a value of 1 representing a perfect positive correlation, and a value of 0 representing no correlation. We used the Pearson correlation coefficient to determine the linear relationship between gene expression levels and the condition (waterlogged vs. control).

### SVM

Although SVM is fundamentally a classification tool, it can be instrumental in feature selection through its use of attribute weights, which are derived from the coefficients of the hyperplane in the SVM model. SVMs operate by finding a hyperplane that best separates the classes in the feature space. In a linear SVM, which is the focus of our analysis, the coefficients of this hyperplane can be interpreted as the importance of each feature (gene) in making the classification decision. Higher absolute values of these coefficients indicate a stronger influence of the corresponding feature on the decision boundary. This property of SVM makes it suitable for feature selection by highlighting genes that are crucial in distinguishing between different classes such as stress vs. control conditions in cucumber plants under waterlogging stress. Combining SVM with other feature selection methods can enhance the robustness and relevance of the selected features (Guyon et al. 2002; Sudha George and Raj 2014). By comparing the features selected by SVM with those identified through other techniques such as LASSO, Relief, Uncertainty, and Correlation, we can achieve a more comprehensive understanding of feature relevance, ensuring that the selected genes are not only statistically significant but also consistently influential across different methods. By focusing on genes identified through the SVM weights and corroborated by other methods, we can streamline the analysis and enhance the interpretability of the model.

The number of retained features for each method was determined based on user-defined thresholds of feature importance scores (> 0.90) to ensure consistency and maintain the highest relevance in the final feature set.

### Least absolute shrinkage and selection operator (LASSO)

LASSO is a type of regression analysis that performs both variable selection and regularization to enhance the prediction accuracy and interpretability of the resulting statistical model. It is particularly useful for datasets with a large number of features, as it can select a subset of the most important features. In the context of gene selection, LASSO helps identify the most relevant genes associated with a particular condition by shrinking the coefficients of less important genes to zero, effectively excluding them from the model.

To identify candidate genes that have a consistent correlation with waterlogging stress, we employed the R package glmnet (version 4.1.4) (Hastie et al. 2021) to train a logistic LASSO regression model using the DEGs (Differentially Expressed Genes) profile. This algorithm performed feature selection by shrinking the coefficients of less important features to zero, thereby retaining only the most significant genes. In our study, the LASSO regression model was fitted to a dataset containing 20,144 genes. Through 10-fold cross-validation, the most suitable value for λ was found. This resulted in the identification of genes with non-zero coefficients, indicating their significant role under waterlogging stress conditions in cucumber.

### Differential Gene correlation analysis (DGCA)

In addition, we conducted a DGCA to investigate further the genes selected by both the attribute weighting algorithms and the LASSO regression method. This analysis aimed to identify changes in correlation patterns between genes under waterlogged versus control conditions, providing deeper insights into the regulatory networks and interactions among the selected genes.

DGCA provides a variety of approaches to calculate and examine differences in gene correlations between different conditions (McKenzie et al. 2016). To investigate the regulatory relationships between genes in control and abiotic stress conditions, we used the DGCA package in R studio. In this method, we transformed correlation coefficients into z-scores and determined p-values to assess differential gene correlation (McKenzie et al. 2016).

To normalize the z-scores, we used the Fisher z-transformation formula:

$$\:z\hspace{0.17em}=\hspace{0.17em}\text{a}\text{t}\text{a}\text{n}\text{h}\left(\text{r}\right)\:=\:\frac{1}{2}{log}_{e}\left(\frac{1+r}{1-r}\right)$$

where ‘r’ is the correlation coefficient of the sample, ‘log_e_’ is the natural logarithm function, and ‘atanh’ is the arc-tangent hyperbolic function. The variance of the z-scores depends on the type of correlation, whether Pearson’s correlation (rp) or Spearman’s correlation (rs) (Fieller et al. 1957). The variance of the normalized distribution can be calculated using the formula, where ‘n’ is the sample size of the correlation:

var(r_p_) = $\:\frac{1}{n-3}$ or var(r_s_) = $\:\frac{1.06}{n-3}$

Next, we computed the difference in z-scores (dz) between the control and abiotic stress conditions:

d*z* = $$\:\frac{({z}_{1}-{z}_{2})}{\sqrt{\left|{S}_{{z}_{1}}^{2}-\:{S}_{{z}_{2}}^{2}\right|}}$$

where $\:{S}_{{z}_{1}}^{2}$ and $\:{S}_{{z}_{2}}^{2}$ represent the variances of the z-scores in the control and abiotic stress conditions, respectively. Using dz, a two-tailed p-value for the standard normal distribution was calculated, and gene pairs were ranked according to their differential correlation values.

### Validation and interpretation of the selected genes

The genes that were significantly paired-correlated between control and waterlogging stress using DGCA analysis were selected for validation. The software R (version 4.1.2) and the R packages ranger (version 0.14.1) were employed for RF classification (Wright and Ziegler 2015). In this study, we selected the RF classifier instead of utilizing SVM. Firstly, SVM does not inherently provide feature importance measures in the way that tree-based models such as RF do. RF provides a measure of feature importance, which can be valuable for understanding which variables contribute most to the predictions. This is particularly useful in domains such as medical research and environmental studies (Adugna et al. 2022). Furthermore, RF typically requires less parameter tuning than SVM, making it easier to use and implement. The decision tree structure of RF also provides some level of interpretability (Adugna et al. 2022).

In contrast, SVM can handle non-linear data through kernel tricks (Sunitha and Raju 2021). RF naturally handles non-linear relationships and interactions between features without requiring explicit transformation (Hong and Lynn 2020). Moreover, RF is generally less prone to overfitting compared to SVM, especially when dealing with high-dimensional data or noisy datasets (Lachaud et al. 2023).

Additionally, RF is an ensemble learning method, which means it combines multiple decision trees to make predictions. This approach often leads to improved accuracy and robustness compared to single-model classifiers like SVM (Natarajan et al. 2023). RF has been successfully applied in various bioinformatics studies for gene selection and classification. For example, Pashaei et al. (2017) showed that the RF classifier performs much faster than the SVM classifier in detecting the splice sites in the human genome.

The number of trees was set to 100 for the model building. Machine learning (ML) models, which are considered as black boxes, can be interpreted using SHAP (SHapley Additive exPlanations) value to explain different ML models (Bingol and Brüschweiler 2015). Therefore, we calculated the SHAP value and the importance of ranking genes from the classification models using “shapviz” version 0.9.1 in R.

In addition, we also employed the Boruta algorithm as a wrapper-based feature selection method to further validate our gene selection strategy. The Boruta algorithm is an all-relevant feature selection method designed to identify the most important features in a dataset by comparing the importance of original attributes with the importance achievable at random. This method iteratively removes features that are statistically less relevant, thereby retaining only the most significant variables (Kursa and Rudnicki 2010).

Using the R package Boruta (version 8.0.0), we trained the model on DEGs matrix. The algorithm performed 100 iterations to ensure robust feature selection, providing a comprehensive measure of attribute importance. The final selection of features was refined using the ‘TentativeRoughFix’ function. This process enabled us to obtain a clear understanding of which feature genes were most relevant to the waterlogging stress condition.

## Results

### Weight by uncertainty

The uncertainty algorithm revealed that four genes exceeded a threshold of 0.90. As shown in Table 2, these genes were LOC101221250, LOC101209599, LOC101216320, and LOC101206172.

### Weight by relief

The Relief algorithm identified four genes with values greater than 0.90. As depicted in Table 2, these genes were LOC101213801, LOC101205971, LOC101214385, and LOC101203449.

### Weight by correlation

The Correlation algorithm revealed that twenty-one genes had values exceeding 0.90. As shown in Table 2, these genes were LOC101213801, LOC101210665, LOC101205805, LOC101217277, LOC101213872, LOC101222503, LOC101206239, LOC101206201, LOC101212424, LOC101212625, LOC101203084, LOC101204590, LOC101210747, LOC101214385, LOC101213580, LOC116403322, LOC101205431, LOC101210491, LOC105435194, LOC101204309, and LOC101205898.

### Weight by SVM

The SVM algorithm indicated that ten genes had values surpassing 0.90. As mentioned in Table 2, these genes were WRKY34, LOC105435136, LOC101222803, LOC101213580, LOC101212625, LOC101206142, LOC101223041, LOC101202985, LOC101207749 and LOC116406191.

**Table 2:** List of genes identified by four attribute weighting algorithms, including uncertainty, relief, correlation, and SVM, with a cut-off value of 0.90

Attribute weighting algorithm	Gene symbol	Weight
**Correlation**	LOC101213801	1
	LOC101210665	0.981792
	LOC101205805	0.976468
	LOC101217277	0.965222
	LOC101213872	0.961903
	LOC101222503	0.95507
	LOC101206239	0.950225
	LOC101206201	0.947782
	LOC101212424	0.946504
	LOC101212625	0.946377
	LOC101203084	0.936201
	LOC101204590	0.929242
	LOC101210747	0.921718
	LOC101214385	0.919868
	LOC101213580	0.9143
	LOC116403322	0.91381
	LOC101205431	0.913398
	LOC101210491	0.903276
	LOC105435194	0.902187
	LOC101204309	0.901909
	LOC101205898	0.900742
**SVM**	WRKY34	1
	LOC105435136	0.955122
	LOC101222803	0.929876
	LOC101213580	0.927133
	LOC101212625	0.926384
	LOC101206142	0.922183
	LOC101223041	0.908447
	LOC101202985	0.908386
	LOC101207749	0.905523
	LOC116406191	0.902036
**Uncertainty**	LOC101221250	1
	LOC101209599	0.956375
	LOC101216320	0.931761
	LOC101206172	0.910819
**Relief**	LOC101213801	1
	LOC101205971	0.964735
	LOC101214385	0.925444
	LOC101203449	0.924483

### LASSO gene selection

**Fig. 2:**
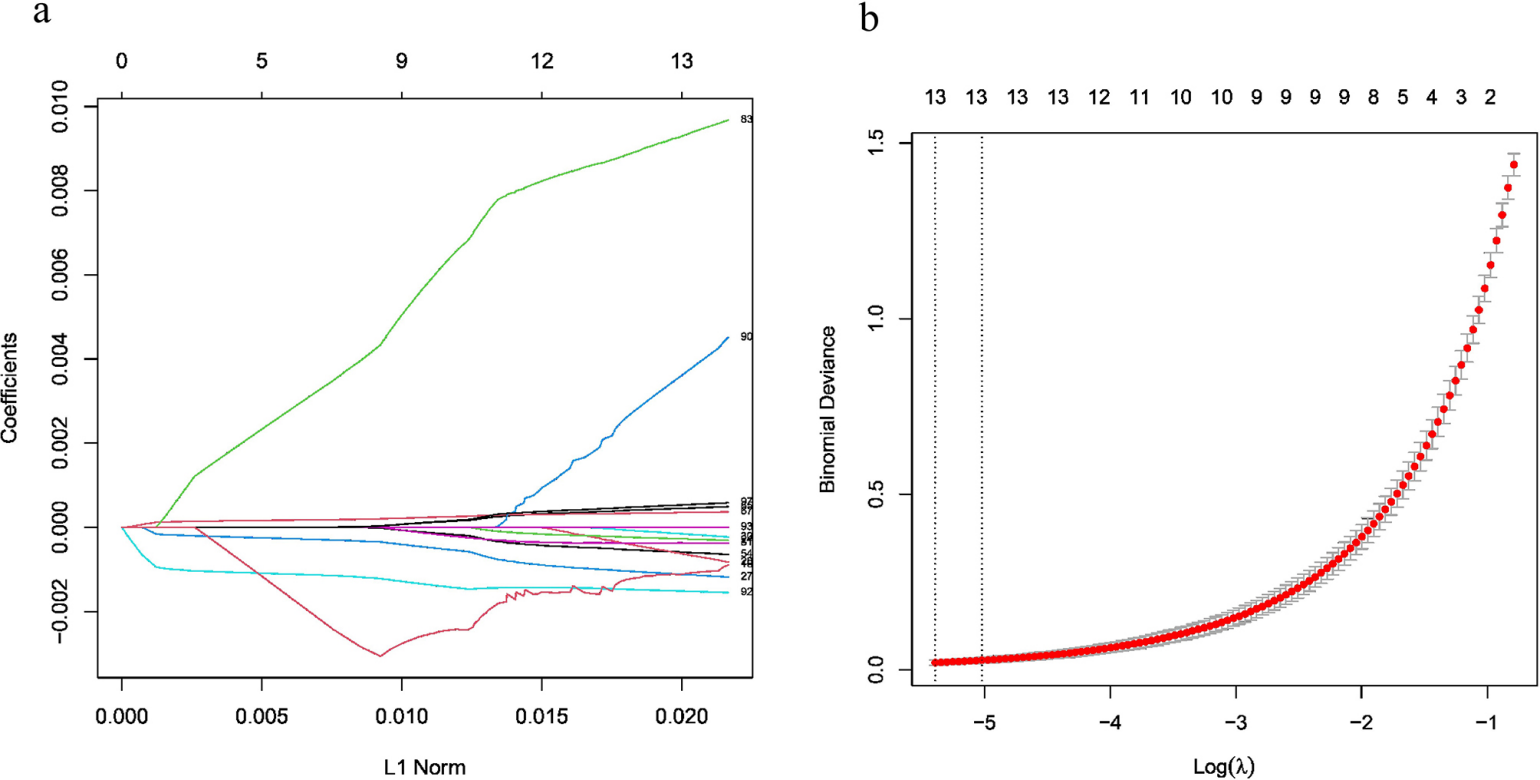
Feature selection using the LASSO logistic regression model by 10-fold cross-validation at lambda.1se. (**a**) The path of variable coefficient against the L1 Norm of the total coefficient vector as *λ* varies with the number of` non-zero coefficients represented on the axis above (**b**) LASSO coefficients of 13 significant genes in waterlogging in cucumber (vertical lines related to lambda.1se)

The LASSO regression model was fitted to a dataset containing 20,144 genes (Fig. 2a). Through 10-fold cross-validation (Fig. 2b), the most suitable value for λ was found to be 0.03858. Subsequently, 13 genes with non-zero coefficients were identified under waterlogging stress conditions in cucumber. These genes include LOC101212625, LOC101213580, LOC101213801, LOC101222503, LOC101205805, LOC101205000, LOC101203084, LOC101214385, LOC101210491, LOC101206239, LOC101210665, LOC101205431 and LOC101206142.

### Differential Gene correlation analysis (DGCA)

In this study, a pairwise analysis was performed on cucumber plants subjected to both control and waterlogging stress conditions. We focused on examining the variation in the correlation between each pair of genes, considering a total of 36 genes selected using LASSO regression and attribute weighting algorithms (supplementary file, sheet S2). This resulted in a total of 630 pairwise comparisons (Supplementary file, sheet S3). The p-values for 117 differential gene correlation (DGC) pairwise comparisons were below 0.01, indicating a significant alteration in the correlation between genes under control and waterlogging stress conditions. For further investigation, we provided a list of the top ten differential gene pairs under both control and waterlogged stress conditions (Table 3).

**Table 3:** Top ten Differential Gene Correlation Analysis (DGCA) comparisons from a collection of 36 genes selected using LASSO regression and attribute weighting algorithms. The first two columns show the ID of paired genes, the columns third and fourth are the correlation and *p*-value of the pair genes under control, the fifth and sixth columns show the correlation and *p*-value of the paired genes under waterlogging stress, the seventh column shows the change in Z-score, indicating the change in the correlation between gene pairs, and the eighth column shows the classes of differentially correlated gene pairs

Gene1	Gene2	control_cor	control_pVal	waterlogging_cor	waterlogging_pVal	zScoreDiff	pValDiff	Classes
LOC101209599	LOC101221250	-0.00583	0.981689	0.985479	9.50E-14	6.750255	1.48E-11	0/+
LOC101209599	LOC101216320	-0.28072	0.259164	0.971161	2.20E-11	6.574823	4.87E-11	0/+
LOC101205971	LOC105435136	-0.71511	0.00085	0.807402	5.10E-05	5.524036	3.31E-08	-/+
LOC101204590	LOC101207749	0.708786	0.000991	-0.8021	6.22E-05	-5.44772	5.10E-08	+/-
LOC101216320	LOC101221250	0.392644	0.107014	0.983571	2.54E-13	5.427623	5.71E-08	0/+
LOC101205805	LOC101217277	-0.46192	0.053628	0.88249	1.26E-06	5.166829	2.38E-07	0/+
LOC101206172	LOC101209599	0.010636	0.966589	0.950915	1.45E-09	5.013351	5.35E-07	0/+
LOC101203084	LOC101206142	-0.36789	0.133089	0.893961	5.74E-07	5.004244	5.61E-07	0/+
LOC101204590	LOC101221250	-0.21779	0.385305	0.916726	8.93E-08	4.900632	9.55E-07	0/+
LOC101203449	LOC101216320	0.922398	5.17E-08	-0.16899	0.502642	-4.86239	1.16E-06	+/0

**Fig. 3:**
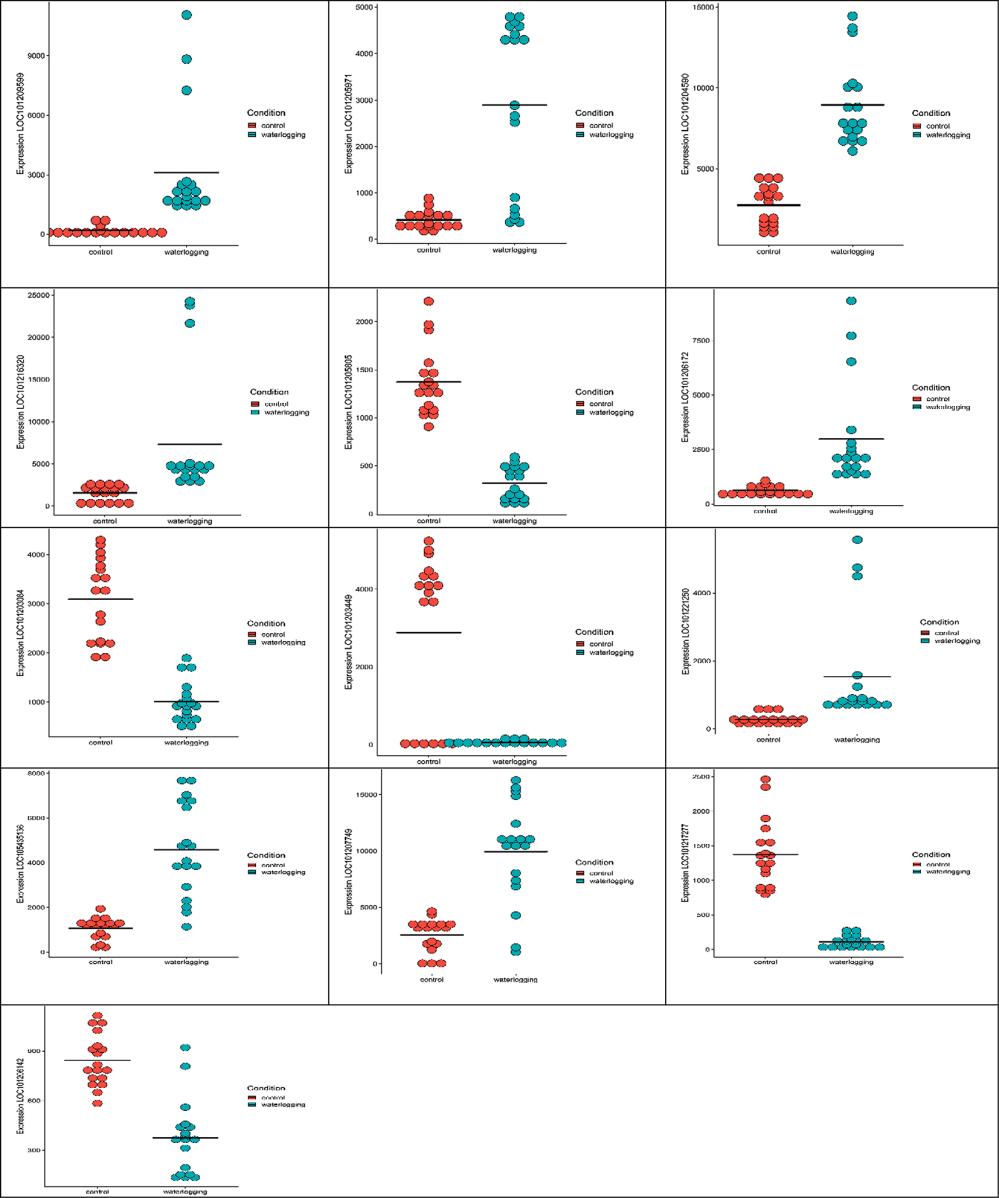
The values of individual genes from the top ten significantly correlated gene pairs identified by Differential Gene Correlation Analysis (DGCA) across conditions are plotted

The values of individual genes from the top ten significantly correlated gene pairs identified by differential gene correlation analysis (DGCA) across conditions are plotted in Fig. 3.

**Fig. 4:**
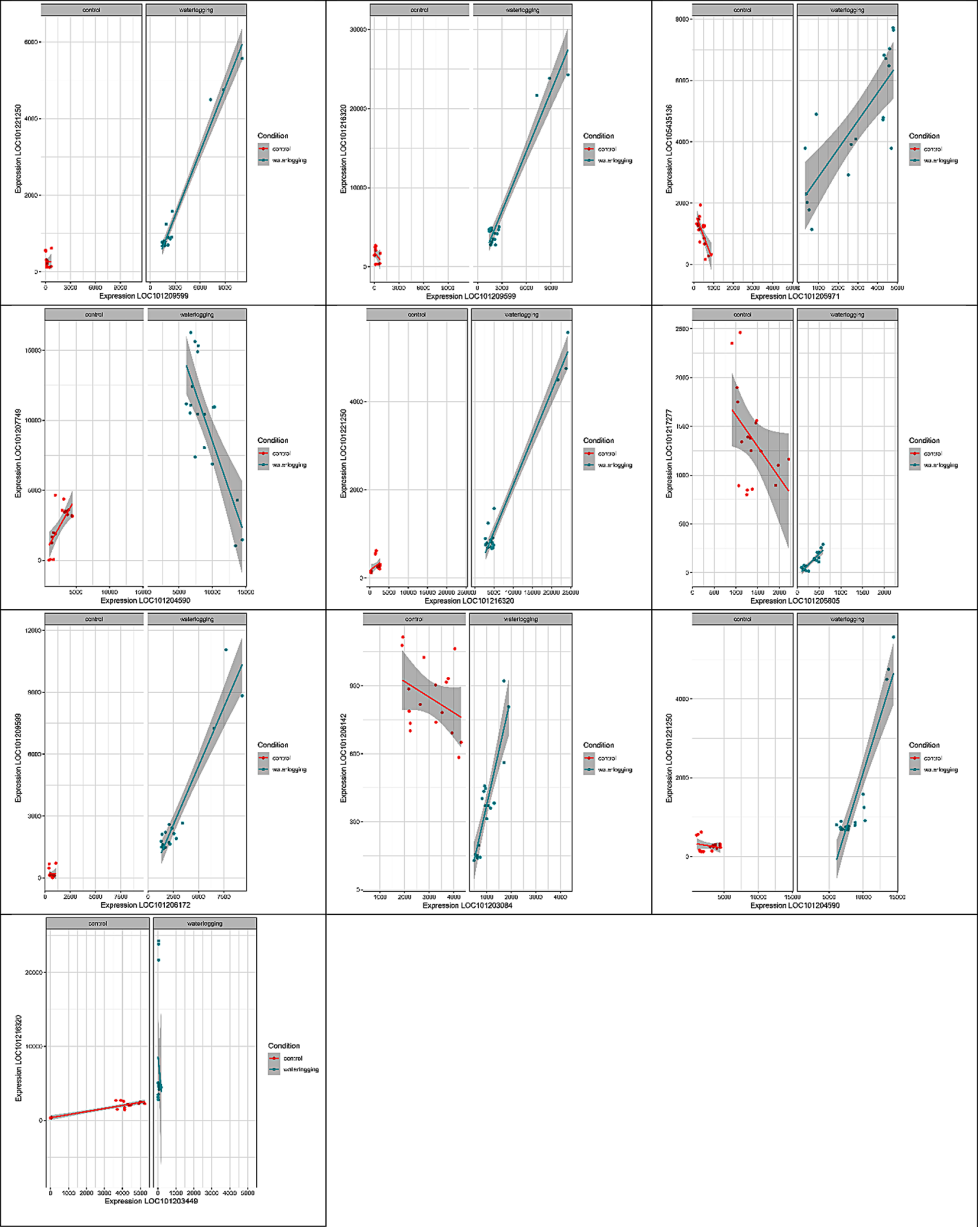
The top ten significantly correlated pairs of genes (*p* < 0.001) were identified through Differential Gene Correlation Analysis (DGCA). The X and Y axes indicate the gene expression values, and each point represents one sample. Colored lines and shaded areas represent the linear regression lines and their respective 95% confidence intervals for each control and stress condition

The differentially correlated gene pairs were then grouped into four categories. In the control condition, seven gene pairs showed no correlation, whereas they showed a positive correlation in the waterlogging stress condition (0/+). In the control condition, there was a positive correlation between one pair of genes, but this correlation was not observed in the waterlogging stress condition (+/0). One pair of genes showed a negative correlation in the control condition, but a positive correlation in the waterlogging stress condition (-/+). Another pair of genes showed a positive correlation in the control condition but exhibited a negative correlation in the waterlogged stress condition (+/-) (Fig. 4).

#### Validation and interpretation of the selected genes

**Fig. 5:**
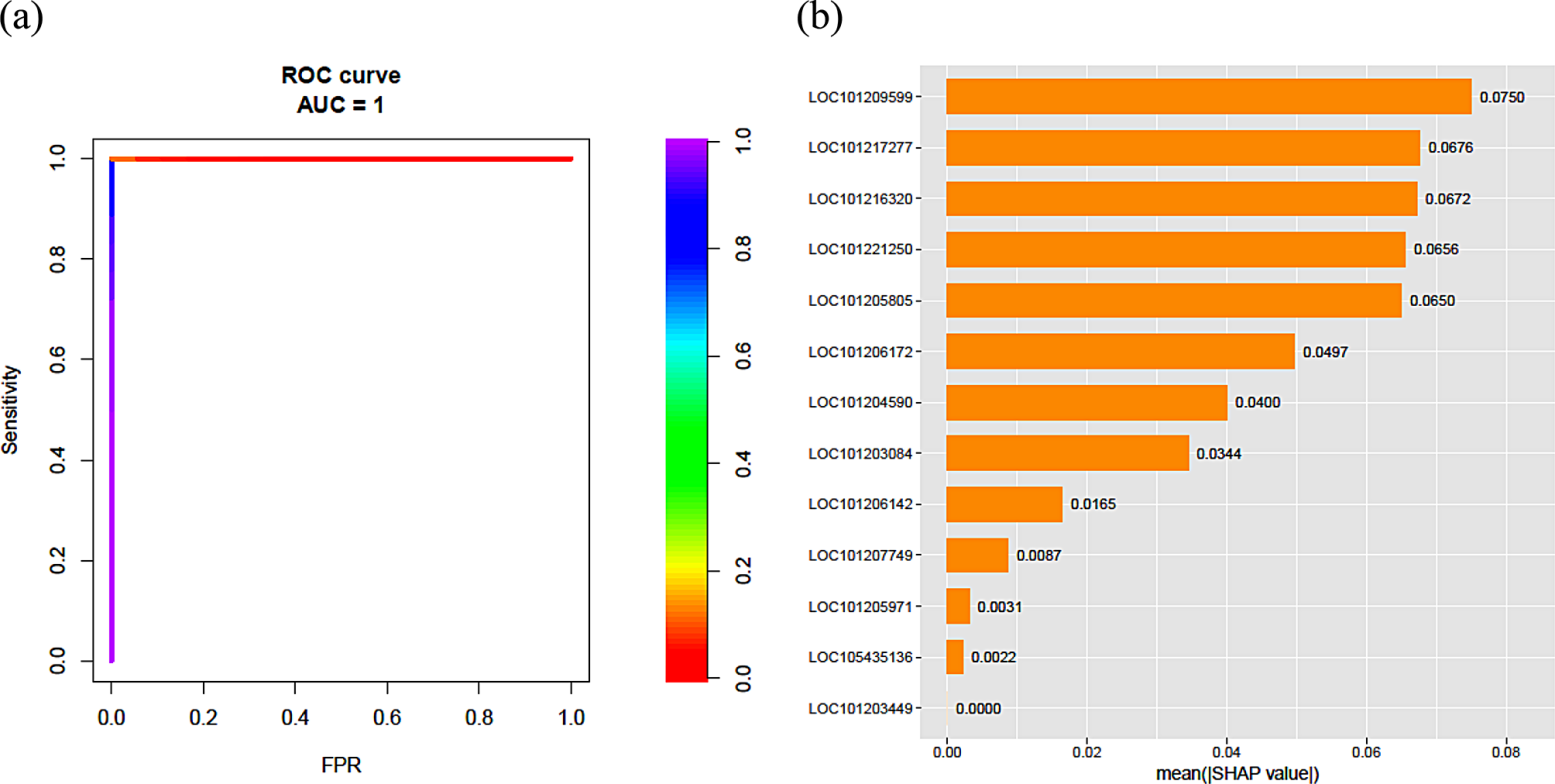
Genes obtained by LASSO regression and value attribute weighting algorithms (13 genes) were used to validate the effectiveness of gene selection using the Random Forest model (**a**). The importance ranking of the 13 selected genes according to the mean (|SHAP value|) and SHAP value of the features is shown (**b**)

We implemented RF, a highly efficient machine learning algorithm, to construct the classification model. The accuracy and area under the curve (AUC) of the RF model were 100% and 1.0, respectively, as shown in the ROC (Fig. 5a). The out-of-bag (OOB) prediction error was estimated to be 0.0017. To visually illustrate how the selected genes affect the model, we used SHAP to differentiate between normal and waterlogged stress in cucumber. Figure 5b illustrates the average absolute SHAP value and significance of the 13 genes in our model. The vertical axis of the gene ranking represents the significance of the genes within the model. The SHAP value, shown on the x-axis, is an index used to quantify the impact of a particular gene within the model.

LOC101209599 followed by LOC101217277, LOC101216320, LOC101221250, and LOC101205805 were the most important genes in the model and may be associated with waterlogging response in cucumber. On the other hand, LOC101203449 was shown to have no effect on the model. Also, genes LOC105435136 and LOC101205971, with low SHAP values have low contribution in the model.

The Boruta algorithm confirmed 106 genes as important in the waterlogging stress response (supplementary file, sheet S4). Examining the overlap between these 106 genes and the 36 genes identified by the four feature weighting algorithms and LASSO, we found that 21 genes were common (supplementary file, sheet S5). Additionally, eight of the 106 genes overlapped with 13 genes identified by the four feature weighting algorithms, LASSO, and the gene correlation analysis (DGCA) (supplementary file, sheet S6).

## Discussion

Waterlogging is a widespread abiotic stress that poses a significant threat to the productivity of crops, including cucumber (*Cucumis sativus*), an important agricultural crop. With the frequency and severity of waterlogging expected to increase with climate change, understanding the response of cucumber to this stress is critical to ensure crop resilience and sustainability. Analysis of sequencing data yields a large number of genes, making it difficult to relate these high-dimensional datasets to specific biological phenomena and their underlying mechanisms. To address this problem, we used the LASSO method, which combines ridge regression and feature selection, to identify a subset of informative genes associated with waterlogging stress in cucumber plants.

Looking at the regulatory relationships between genes is also very important for building models that can predict what will happen in biological systems. Using DGCA, we have been able to learn more about how gene-gene relationships change in different situations of interest. This method not only detects crucial changes in gene regulatory relationships, but also facilitates the investigation of unexplored signaling pathways, biomarkers and targets in complex biological systems. In our research, we focused on genes identified using LASSO regression and weighting techniques and applied DGCA to investigate differences in gene correlations between control and waterlogging stress conditions. The results revealed 13 genes that showed a significant change in correlation with each other under waterlogging stress compared to the control condition (Table 4, supplementary file, sheet S7).

**Table 4:** List of 13 genes that are included in the top ten gene pairs with the most significant changes in the correlation between the two conditions

Gene symbol	Ensembl gene ID	Entrez gene ID	Gene description
LOC101203084	Csa_6G190460	101,203,084	U-box domain-containing protein 33
LOC101205805	Csa_7G329330	101,205,805	auxin response factor 18
LOC101206142	Csa_6G495030	101,206,142	formamidase
LOC101217277	Csa_3G838720	101,217,277	probable galactinol–sucrose galactosyltransferase 5-like
LOC101204590	Csa_1G569360	101,204,590	uncharacterized
LOC101216320	Csa_1G666980	101,216,320	kelch repeat-containing protein At3g27220
LOC101206172	Csa_003992	101,206,172	ABC transporter G family member 24-like
LOC101209599	Csa_014188	101,209,599	transcription factor MYB62
LOC101221250	Csa_1G481730	101,221,250	protein ALP1-like
LOC101203449	Csa_1G569280	101,203,449	bidirectional sugar transporter SWEET16
LOC101205971	Csa_4G335240	101,205,971	acid phosphatase 1
LOC105435136	Csa_001144	105,435,136	AAA-ATPase At3g28580
LOC101207749	Csa_1G589650	101,207,749	IQ domain-containing protein IQM1

Out of ten gene pairs, seven showed a decrease in correlation after waterlogging, while one showed a decrease in correlation. Two gene pairs are inversely correlated in the control compared to the stress condition. As shown in Table 3, three genes, including protein ALP1-like, Kelch repeat-containing protein, and ABC transporter G family member 24-like were found to have a positive correlation with MYB62 in the waterlogging stress condition but not in the control condition.

MYB62, which is a member of the MYB gene family, stands out among the genes identified. It is widely recognized for its involvement in plant development and responses to various stresses such as drought and waterlogging (Borrego-Benjumea et al. 2020; Juntawong et al. 2014; Mmadi et al. 2017; Wang et al. 2023). Previous studies have suggested a role for MYB genes in regulating stress responses in various plant species, and a study on sesame in particular highlighted their potential to improve stress tolerance in crops (Mmadi et al. 2017). In cucumber, MYB transcription factors were shown to be differentially expressed under waterlogging stress (Qi et al. 2012). Despite the evidence supporting the involvement of MYB genes in stress responses, there is currently no direct information on the relationship between MYB62 and waterlogging in cucumber. The results of our investigation suggest that MYB62 may have an influence on the response of cucumber to waterlogging stress. However, further research is required to gain a full understanding of its precise role and regulatory mechanisms in this particular environment.

With regard to the ALP1-like protein, despite the limited information available, we can infer a possible role based on its similarity to the ANTAGONIST OF LIKE HETEROCHROMATIN PROTEIN 1 (ALP1) gene. ALP1 has been shown to disrupt gene silencing activity by antagonizing the function of POLYCOMB REPRESSIVE COMPLEX 2 (PRC2), a protein complex involved in the repression of gene expression through histone methylation (Liang et al. 2015). Additionally, PRC2, by increasing H3K27me3 levels in the promoter of ABI4, represses the expression of ABI4 (Godwin and Farrona 2022). We hypothesize that up-regulation of ALP1-like protein under waterlogged stress conditions could result in reduced PRC2 activity, which would subsequently lead to increased expression of specific genes, possibly including ABI4, and influencing lateral root development. Cucumber’s weak water absorption ability and less established root system make it susceptible to waterlogging stress (Pan et al. 2021), further emphasizing the importance of understanding the role of ALP1-like protein and its potential contribution to stress tolerance.

Kelch repeat-containing proteins are a subfamily of F-box proteins that are found almost exclusively in plants (ul Hassan et al. 2015; Wei et al. 2021). The role of Kelch repeat-containing protein in waterlogging is not fully understood. However, a study on soybean roots under waterlogging stress revealed that a Kelch repeat-containing F-box family protein was among the differentially expressed genes (Alam et al. 2010). Another study on sugarcane found that a Kelch repeat-containing F-box-like protein was involved in protein degradation in response to waterlogging (Khan et al. 2014). Kelch repeat F-box (KFB) proteins, including Kelch repeat-containing proteins, are involved in ubiquitin-mediated protein degradation through selective binding of target proteins (Tang et al. 2022). Waterlogging can accelerate the degradation of proteins and chlorophyll in leaves, which reduces the capacity of leaves to photosynthesize and can lead to leaf senescence and yellowing (Pan et al. 2021; Stieger and Feller 1994). Therefore, it is possible that the Kelch repeat-containing protein plays a role in protein degradation and other cellular processes in cucumber in response to waterlogging stress.

ABC transporters, particularly the ABCG subfamily, have been identified as crucial contributors to maintaining plant homeostasis and responding to abiotic stresses (Dahuja et al. 2021; Wu et al. 2022). Our results suggest that ABC transporter G family member 24-like is among the genes with altered correlations under waterlogging stress. Although its specific function in the response of cucumber to waterlogging is still unclear, we can gain insights from studies in other plant species. For example, mutations in the ABCG5 gene have been shown to affect growth and responses to waterlogging in plants (Do et al. 2021). Further studies are needed to determine the precise role of the ABC transporter G family member 24-like in cucumber under waterlogging stress.

The identified acid phosphatase is an intriguing candidate owing to its potential role in enhancing plant phosphorus acquisition. The increased uptake of phosphorus under waterlogging stress and its involvement in mitigating abiotic stresses, including drought and salinity, in plants has been documented (Bechtaoui et al. 2021; Rubio et al. 1997). This suggests that inorganic phosphate may have a critical function in mitigating waterlogging stress in cucumber, ultimately contributing to stress alleviation.

We identified the AAA-ATPase gene as a potential player in the response to waterlogging stress. Notably, a previous investigation conducted by (Xu et al. 2018) has substantiated the potential role of the AAA-ATPase gene, CsARN6.1, in cucumber in waterlogging tolerance and improving adventitious root formation. Under waterlogged conditions, transgenic cucumber plants carrying the CsARN6.1Asp allele from Zaoer-N exhibited a significant increase in the number of adventitious roots compared to the wild-type cucumbers expressing the allele from Pepino. These findings suggest that the AAA-ATPase gene CsARN6.1 plays a crucial role in promoting adventitious root formation and improving waterlogging tolerance in cucumber (Xu et al. 2018). The validated roles of several identified genes, including AAA-ATPase, further confirm the credibility and robustness of our research findings.

Another gene that was identified in our study is the IQ domain-containing protein IQM1, which exhibited the upregulation in response to waterlogging stress. IQM1, a calmodulin-binding protein, has been reported to be associated with the stomatal movement in Arabidopsis (Zhou et al. 2012). In particular, waterlogging stress has been shown to induce an increase in cytosolic calcium levels in plants (Li et al. 2022). Consequently, the binding of calcium ions to calmodulin induces conformational changes, facilitating its interaction with target proteins involved in various cell signaling events (Tan et al. 2019). Based on these findings, it is plausible to hypothesize that calmodulin-binding proteins, including IQM1, may actively contribute to the cucumber response mechanisms to waterlogging stress.

In our study, the identification of auxin response factor 18 contributes to our understanding of the response of cucumber to waterlogging stress. Notably, previous research by (Qi et al. 2012) highlighted the up-regulation of two additional auxin response factors, auxin response factor 3 and auxin response factor 2, in waterlogged cucumber roots. This suggests that auxin may be a crucial signal mediating the plant defense against waterlogging stress. ARFs (Auxin Response Factors) are known as transcriptional activators of early auxin response genes, and they play a crucial role in regulating lateral root formation in *Arabidopsis thaliana* (Okushima et al. 2007). In our study, reduced expression of galactinol-sucrose galactosyltransferase 5-like protein and auxin response factor 18 was observed under waterlogging stress, accompanied by a positive correlation between these genes. The function of galactinol-sucrose galactosyltransferase 5-like protein (SEED IMBIBITION 1-LIKE; Raffinose synthase 5) is to catalyze the synthesis of raffinose. Additionally, there is evidence that some ARFs can regulate the expression of genes involved in sugar metabolism (Yuan et al. 2019). One study suggests that auxin signaling components, including ARFs, may play a role in regulating the expression of raffinose synthase (Han et al. 2020). These findings highlight the multifaceted involvement of auxin and its associated transcriptional activators, the Auxin Response Factors (ARFs). They may not only serve as a crucial signal mediating the plant’s defense against waterlogging stress but also play a significant role in regulating lateral root formation. ARFs also appear to influence the gene expression related to sugar metabolism, such as galactinol-sucrose galactosyltransferase 5-like protein. When plants experience stress, they reprogram their metabolism and gene expression to divert energy sources from growth-related biosynthetic processes to defense, acclimation, and adaptation. Sugar metabolism is an important component of energy signaling in plants, as sugars serve as important energy sources for growth and development (Baena-González 2010; Nägele et al. 2022). Therefore, regulation of sugar metabolism appears to be a critical factor in enabling cucumber plants to respond and adapt to stressful conditions effectively.

Formamidase, another gene identified in this study, has been suggested to be involved in enhancing abiotic stress tolerance in plants For example, a study in barley showed that when the plant was exposed to heat and drought, it exhibited changes in gene expression, including genes associated with formamidase (Mahalingam et al. 2022). In Arabidopsis, two recently reported formamidase-like proteins, *IAMH1* and *IAMH2*, have been linked to the conversion of formamide to formate, which is involved in responses to abiotic stress (Moya-Cuevas et al. 2021).

The U-box domain-containing protein 33, discovered in this research, functions as an E3 ubiquitin ligase and is involved in the degradation of group VII ethylene response factor (ERFVII) transcription factors, which are associated with hypoxia responses in plants. Decreased expression of the E3 ligase leads to increased expression of hypoxia-associated genes and altered seed germination in waterlogged transgenic plants (Mendiondo et al. 2016). The oxygen sensor reporter protein MCGGAIL-GUS also increases in waterlogged transgenic plants with reduced expression of E3 ligase (Mendiondo et al. 2016). These results suggest that manipulation of E3 ligase expression affects the stability of ERFVII transcription factors and their downstream targets, leading to increased tolerance to waterlogging in barley (Mendiondo et al. 2016). In our study, the expression of U-box domain-containing protein 33 was found to be downregulated under waterlogging. It can be concluded that reducing the expression of U-box domain-containing protein 33, which acts as an E3 ubiquitin ligase, leads to increased expression of genes associated with hypoxia and stress response to waterlogging in cucumber plants.

Regarding AtSWEET16, it served as a fructose/glucose/sucrose uniporter located on the tonoplast membrane and plays a key role in maintaining sugar homeostasis (Guo et al. 2014). Our results showed the down-regulation of the bidirectional sugar transporter SWEET16 under waterlogging stress, which is consistent with previously reported observations. For instance, the expression of SWEET16 was down-regulated under various stress conditions such as cold, osmotic stress, or low nitrogen, as well as in response to the application of glucose, fructose, or sucrose. Under cold stress conditions, the overexpression of AtSWEET16 led to a reduction in fructose concentration in leaves (Guo et al. 2014). Given its critical role in sugar homeostasis, the activity of AtSWEET16 must be tightly regulated to allow optimal development of Arabidopsis under stress conditions. AtSWEET16 overexpressing plants also displayed enhanced freezing tolerance (Klemens et al. 2013).

In summary, the identified genes may regulate various physiological processes such as stress tolerance, root development, nutrient uptake, and sugar metabolism, as well as molecular processes such as gene expression, protein degradation, and calcium signaling. These processes collectively help cucumber to cope with the challenges of waterlogging stress and enable the plant to adapt to stress conditions. However, more investigation is required to elucidate the specific roles of these genes in cucumber’s response to waterlogging stress and their interconnected pathways.

Finally, 13 genes with significant paired correlations between the control group and waterlogged stress conditions were rigorously validated using the RF classification model. The accuracy and area under the curve (AUC) of the RF model were both exceptionally high, at 100% and 1.0, respectively, as shown in the ROC curve (Fig. 5a). To visually illustrate the impact of the selected genes on the model, we employed SHAP values to discriminate between normal and waterlogged stress conditions in cucumber. Notably, genes such as LOC101209599, LOC101217277, and LOC101216320 showed a significant influence on the model’s predictive power, emerging as pivotal players, particularly concerning cucumber response to waterlogging stress. Conversely, genes such as LOC101203449 had a negligible effect. These findings illuminate the molecular intricacies underlying cucumber plant responses to waterlogging stress, shedding light on potential targets for further research and crop improvement approaches.

Overall, our study contributes to the growing body of knowledge on the response of cucumber to waterlogging stress. The application of machine learning to transcriptomic data allowed us to comprehensively explore the molecular landscape of the stress response in cucumber. The candidate genes discovered in this research provide promising avenues for future investigation and strategies to improve crop management. Understanding the underlying processes that govern the tolerance of cucumber plants will contribute to the development of stress-resistant cucumber varieties, thereby improving food security and agricultural sustainability in the face of changing environmental conditions. However, it is imperative to acknowledge the limitations of our study. The datasets used in this analysis may not capture the entire complexity of the cucumber response to waterlogging stress, and additional experiments and validation are required to confirm the roles of the identified genes definitively. Moreover, gene function may be context-dependent, and further research is required to determine the precise processes through which these genes are involved in stress adaptation.

In this study, we used three transcriptomic datasets to investigate the effects of waterlogging stress on cucumber plants. Using machine learning and LASSO logistic regression analyses, we aimed to elucidate the molecular mechanisms behind the plant’s response to waterlogging and identify genes that help it adapt to stress. Using various machine learning techniques, we were able to pinpoint cucumber genes associated with waterlogging stress. The Uncertainty, Relief, SVM, and Correlation algorithms revealed four, four, ten, and twenty-one genes, respectively, with weights greater than 0.90. Additionally, the LASSO algorithm identified thirteen genes associated with waterlogging stress adaptation in cucumber plants. To gain deeper insights into the functional significance of the identified genes, we conducted a differential gene correlation study. This analysis revealed significant changes in the correlation between 13 genes under control and waterlogging stress conditions. These altered correlations indicate the dynamic nature of gene interactions in response to stress, emphasizing the importance of studying gene networks in the context of stress adaptation. Furthermore, the efficacy of these 13 genes was demonstrated using the RF model and the SHAP value. The RF model performed perfectly, considering an accuracy of 100% and area under the curve (AUC) of 1. The model’s utilization of the 13 genes highlighted the significant impact of LOC101209599, LOC101217277, and LOC101216320. In addition, we use the Boruta algorithm as a wrapper-based feature selection method to further validate our gene selection strategy. The Boruta algorithm confirmed 106 genes as important in the waterlogging stress response. Examining the overlap between these 106 genes and the 36 genes identified by the four feature weighting algorithms and LASSO, we found that 21 genes were common. Examining the overlap between these 106 genes and the 13 genes identified by the four feature weighting algorithms, LASSO and DGCA, we found that eight genes were common. These results indicate that the filter-based (Uncertainty, Relief, Correlation, SVM) and embedded (LASSO) methods also performed well in identifying genes related to waterlogging stress response in cucumber, similar to the wrapper-based Boruta approach.

Our results not only demonstrate the complexity of cucumber’s response to waterlogging but also provide insight into potential key players that contribute to stress tolerance. In conclusion, our study offers crucial information about the molecular basis of the cucumber’s response to waterlogging stress. It highlights the usefulness of integrating transcriptomic data and machine learning techniques to unravel complex stress responses in plants. The identified candidate genes hold promising potential for cucumber improvement, and future research should focus on validating their functions and exploring their potential applications in breeding stress-tolerant cucumber varieties.

## Conclusions

Overall, the innovative integration of multiple feature selection methods, the meta-analysis approach, and the depth of biological insights (DGCA) obtained constitute the key novelty of this work. Our study presents an innovative integration of multiple feature selection methods from different categories to analyze the response cucumber to waterlogging stress comprehensively. By combining these different methods, we leverage their respective strengths to achieve a more robust and comprehensive gene selection process, thereby reducing bias. Furthermore, we performed a meta-analysis of three independent transcriptome datasets (PRJNA799460, PRJNA844418, PRJNA678740), which improves the generalizability and robustness of our results by accounting for variability under different experimental conditions. Applying DGCA to the genes selected using this multi-method approach revealed new insights into the regulatory networks and interactions critical for the adaptation of cucumbers to waterlogging stress, enabling a deeper understanding beyond merely listing differentially expressed genes. The significance of the 13 identified genes was validated using the RF model, achieving an accuracy of 100% and an AUC score of 1.0. SHAP values were used to interpret the model, highlighting the functional importance of specific genes such as LOC101209599, LOC101217277, and LOC101216320 in waterlogging response. Moreover, the Boruta algorithm applied as a wrapper-based feature selection method underscores the robustness and reliability of our gene selection strategy. Interestingly the genes LOC101209599, LOC101217277, and LOC101216320 were among genes identified by multiple feature selection methods from different categories (filtering, wrapper, and embedded). These genes represent valuable targets for future breeding programs to improve stress tolerance in cucumbers.

### Electronic supplementary material

Below is the link to the electronic supplementary material.

Supplementary Material 1

## Data Availability

All data has been provided with the manuscript. If any additional information is required, then the corresponding author can be contacted.
